# ﻿*Zhangixalusthaoae* sp. nov., a new green treefrog species from Vietnam (Anura, Rhacophoridae)

**DOI:** 10.3897/zookeys.1197.104851

**Published:** 2024-04-08

**Authors:** Tao Thien Nguyen, Huy Hoang Nguyen, Hoa Thi Ninh, Linh Tu Hoang Le, Hai Tuan Bui, Nikolai Orlov, Chung Van Hoang, Thomas Ziegler

**Affiliations:** 1 Institute of Genome Research, Vietnam Academy of Science and Technology, 18 Hoang Quoc Viet Road, Hanoi, Vietnam Vietnam Academy of Science and Technology Hanoi Vietnam; 2 Department of Herpetology, Zoological Institute, Russian Academy of Sciences, 199034, St. Petersburg, Russia Zoological Institute, Russian Academy of Sciences St. Petersburg Russia; 3 Forest Resources and Environment Centre, 300 Ngoc Hoi Road, Thanh Tri, Hanoi, Vietnam Forest Resources and Environment Centre Hanoi Vietnam; 4 AG Zoologischer Garten Köln, Riehler Strasse 173, D-50735 Cologne, Germany AG Zoologischer Garten Köln Cologne Germany; 5 Institute of Zoology, University of Cologne, Zülpicher Straße 47b, D-50674 Cologne, Germany University of Cologne Cologne Germany

**Keywords:** 16S rRNA gene, Lao Cai Province, molecular phylogeny, morphology, new species

## Abstract

We describe a new treefrog species from Lao Cai Province, northwestern Vietnam. The new species is assigned to the genus *Zhangixalus* based on a combination of the following morphological characters: (1) dorsum green, smooth; body size medium (SVL 30.1–32.2 in males); (2) fingers webbed; tips of digits expanded into large disks, bearing circum-marginal grooves; (3) absence of dermal folds along limbs; (4) absence of supracloacal fold and tarsal projection. The new species can be distinguished from its congeners by: (1) dorsal surface of the head and body green without spots; (2) axilla and groin cream with a black blotch; (3) ventral cream without spot; (4) chin creamy with grey marbling; anterior part of the thigh and ventral surface of tibia orange without spots; posterior parts of thigh orange with a large black blotch; (5) ventral side of webbing orange with some grey pattern (6) iris red-bronze, pupils black; (7) finger webbing formula I1¼-1¼II1-2III1-1IV, toe webbing formula I½-½II0-1½III¼-1¾IV1¾-½V. Phylogenetically, the new species is nested in the same subclade as *Z.jodiae*, *Z.pinglongensis*, and *Z.yaoshanensis*, with genetic distances ranging from 3.23% to 4.68%. The new species can be found in evergreen montane tropical forests at an elevation of about 1,883 m a.s.l. This new discovery brings the number of known genus *Zhangixalus* species to 42 and the number of species reported from Vietnam to 10.

## ﻿Introduction

The genus *Zhangixalus* Li, Jiang, Ren & Jiang, 2019 currently contains 42 species, with a wide distribution in northeastern India, Nepal, Bhutan, southern China, Myanmar, northern Thailand, Laos, northern Vietnam, Taiwan, and Japan, and south to Indonesia, Brunei, and Malaysia (Frost 2024). Brakels et al. (2023) revealed two major clades in the genus *Zhangixalus* in their phylogenetic analysis of the group; these include a continental Southeast Asian and East Asian species group and a Sundaland group. Brakels et al. (2023) also included six species in the *Z.chenfui* group, distributed in China, Vietnam, and Laos, including *Zhangixaluschenfui* (Liu, 1945), *Z.jodiae* (Nguyen, Ninh, Orlov, Nguyen & Ziegler, 2020), *Z.melanoleucus* Brakels, Nguyen, Pawangkhanant, Idiiatullina, Lorphengsy, Suwannapoom & Poyarkov, 2023, *Z.nigropunctatus* (Liu, Hu & Yang, 1962), *Z.pinglongensis* (Mo, Chen, Liao & Zhou, 2016), and *Z.yaoshanensis* (Liu & Hu, 1962).

Among the nine *Zhangixalus* species reported from Vietnam, *Z.dennysi* (Blanford, 1881) has been reported from southeastern China to northeastern Vietnam, *Z.dorsoviridis* (Bourret, 1937) from northwestern Vietnam and southern China; *Z.duboisi* (Ohler, Marquis, Swan & Grosjean, 2000) from the Hoang Lien range in Vietnam and China; *Z.feae* (Boulenger, 1893) from Myanmar to the Tay Nguyen Plateau, Vietnam; *Z.franki* Ninh, Nguyen, Orlov, Nguyen & Ziegler, 2020 exclusively from Ha Giang Province, Vietnam; *Z.hungfuensis* (Liu & Hu, 1961) from Lao Cai Province, Vietnam, and Sichuan and Guangxi provinces, China; *Z.jodiae* Nguyen, Ninh, Orlov, Nguyen & Ziegler, 2020 from Ha Giang Province, Vietnam; *Z.pachyproctus* Yu, Hui, Hou, Wu, Rao & Yang, 2019 from northern Vietnam, Yunnan Province, China, and Prachuap Khiri Khan, Thailand; and *Z.puerensis* (He, 1999) from Lao Cai and Ha Giang provinces, Vietnam, and Yunnan, China (Frost 2024).

During our 2019 fieldwork in Y Ty Commune, Bat Xat District, Lao Cai Province, Vietnam, we collected a series of tree frogs that morphologically resembled *Z.yaoshanensis*, a species known from Guangxi Province, China (Liu and Hu 1962). However, the newly discovered population from Vietnam differs from *Z.yaoshanensis* by the following distinct morphological characteristics: size medium (SVL 30.1–32.2 mm in males); dorsum green without spots, venter cream without spots; flank, axilla, and posterior thigh cream with large black blotches. Furthermore, our phylogenetic analysis shows that the Lao Cai Province population is nested in the same subclade with *Z.jodiae*, *Z.pinglongensis*, and *Z.yaoshanensis* in the *Z.chenfui* group.

The pairwise distance from the newly collected species to the congeners of *Zhangixalus* species ranges from 3.23% (compared to *Z.pinglongensis*) to 10.83% (compared to *Z.smaragdinus*). Meanwhile, the genetic distance among species of *Zhangixalus* ranges from 0.49% (*Z.dugritei* and *Z.hui*) to 11.89% (*Z.pachyproctus* and *Z.yaoshanensis*). This demonstrates that the treefrog from Y Ty Commune is a distinct taxon, with a genetic distance of at least 3.23% from other *Zhangixalus* species.

Based on the morphological characters and molecular information, we describe here the unnamed *Zhangixalus* species from Lao Cai Province, Vietnam, as a new species.

## ﻿Materials and methods

### ﻿Repositories, Institutional acronyms, or Institutional abbreviations

**IEBR** Institute of Ecology and Biological Resources

**ROM** Royal Ontario Museum

### ﻿Sampling

The field survey was conducted from 25 April to 1 May 2019 by C.V. Hoang and A.M. Luong in Y Ty Commune, Bat Xat District, Lao Cai Province, northwestern Vietnam. Geographic coordinates and elevations were obtained using a Garmin GPSMAP 76CSX (using the WGS84 datum). After the frogs were photographed alive, three specimens of the new species (IEBR A 5136, IEBR A 5137 and IEBR A 5138) (Table 1) were anaesthetized and euthanized in a closed vessel with a piece of cotton wool containing ethyl acetate (Simmons 2002), fixed in 80% ethanol for 5 h, and then transferred to 70% ethanol for permanent storage. Liver-tissue samples were preserved separately in 96% ethanol before fixation and subsequently deposited in the collection of IEBR.

**Table 1.** Samples of *Zhangixalus* and other species were used for DNA analysis in this study.

No.	Scientific name	Voucher	Locality	GenBank no.	Source
1.	*Rhacophoruskio*	VN.2018.84	Kon Tum, Vietnam		This study
2.	*R.kio*	VN.2018.83	Kon Tum, Vietnam		This study
3.	*Zhangixaluschenfui*	RaoZT0806013	Zhaotong, Yunnan, China	JX219431	Li et al. 2012b
4.	*Z.chenfui*	Li05	Mt. Omei, Sichuan, China	JX219432	Li et al. 2012b
5.	*Z.dennysi*	ML.2019.1	Vinh Phuc, Vietnam		This study
6.	*Z.dennysi*	ML.2019.2	Vinh Phuc, Vietnam		This study
7.	*Z.dorsoviridis*	YN080446	Jinping, Yunnan	JX219425	Li et al. 2012b
8.	*Z.dorsoviridis*	Yt.2018.16	Lao Cai, Vietnam		This study
9.	*Z.dorsoviridis*	YT 2018 6	Lao Cai, Vietnam		This study
10.	*Z.duboisi*	VNMN7079	Ha Giang, Vietnam		This study
11.	*Z.duboisi*	VNMN010243	Lai Chau, Vietnam		This study
12.	*Z.dugritei*	LJT 051002	Sichuan, China	JN688872	Li et al. 2012a
13.	*Z.dugritei*	LJT 051008	Sichuan, China	JN688873	Li et al. 2012a
14.	*Z.feae*	HB.2014.28	Hoa Binh, Viet Nam		This study
15.	*Z.feae*	VNMN05859	Lai Chau Viet Nam		This study
16.	*Z.franki*	VNMN 011686	Ha Giang, Vietnam	LC548745	Ninh et al. 2020
17.	*Z.franki*	VNMN 011687	Ha Giang, Vietnam	LC548746	Ninh et al. 2020
18. L	*Z.hongchibaensis*	CIB 097696	Chongqing, China	JN688882	Li et al. 2012a
19.	*Z.hongchibaensis*	CIB 097687	Chongqing, China	JN688883	Li et al. 2012a
20.	*Z.hui*	SCUM 0504111 L	Sichuan, China	JN688877	Li et al. 2012a
21.	*Z.hui*	SCUMLi 01	Sichuan, China	JN688878	Li et al. 2012a
22.	*Z.hungfuensis*	SCUM 060425L	Sichuan, China	EU215538	Li et al. 2008
23.	*Z.hungfuensis*	SCUM 060424 L	Sichuan, China	JN688879	Li et al. 2012a
24.	*Z.jodiae*	VNMN 07121	Ha Giang, Vietnam	LC545594	Nguyen et al. 2020
25.	*Z.jodiae*	VNMN 07122	Ha Giang, Vietnam	LC545595	Nguyen et al. 2020
26.	*Z.lishuiensis*	YPX47794	Lishui, Zhejiang, China	KY653719	Liu et al. 2017
27.	*Z.lishuiensis*	YPX47792	Lishui, Zhejiang, China	KY653720	Liu et al. 2017
28.	*Z.melanoleucus*	BEI 01010	Phou Samsoum Mt., Xiengkhoang, Laos	OQ305233	Brakels et al. 2023
29.	*Z.melanoleucus*	BEI 01011	Phou Samsoum Mt., Xiengkhoang, Laos	OQ305235	Brakels et al. 2023
30.	*Z.melanoleucus*	ZMMU A7781	Phou Samsoum Mt., Xiengkhoang, Laos	OQ305234	Brakels et al. 2023
31.	*Z.nigropunctatus*	GZ070658	Weining, Guizhou, China	JX219430	Li et al. 2012b
32.	*Z.nigropunctatus*	Li06	Weining, Guizhou, China	JX219433	Li et al. 2012b
33.	*Z.omeimontis*	Li02	Sichuan, China	JX219420	Li et al. 2012b
34.	*Z.omeimontis*	RaoZT0806010	Sichuan, China	JX219419	Li et al. 2012b
35.	*Z.pachyproctus*	TQ.2018.72	Tuyen Quang, Viet Nam		This study
36.	*Z.pachyproctus*	VNMN:1299	Nghe An, Vietnam	LC545592	This study
37.	*Z.pinglongensis*	NHMG201002011	Guangxi, China	KU170684	Mo et al. 2016
38.	*Z.pinglongensis*	NHMG201002003	Guangxi, China	KU170683	Mo et al. 2016
39.	*Z.puerensis*	VNMN 010284	Lai Chau, Viet Nam		This study
40.	*Z.puerensis*	SCUM 060648 L	Yunnan, ChinaYunnan, China	JN688884	Li et al. 2012a
41.	*Z.schlegelii*	KUHE 44531	Okayama, Japan	LC369670	Matsui et al. 2018
42.	*Z.schlegelii*	Genbank	Hiroshima, Japan	NC007178	Sano et al. 2005
43.	*Z.smaragdinus*	RAO6241	Tibet, China	JX219411	Li et al. 2012b
44.	*Z.smaragdinus*	CAS 224708	Nagmung, Putao District, Kachin, Myanmar	MN613214	Yu et al. 2019
45.	*Z.wui*	CIB 097685	Hubei, China	JN688881	Li et al. 2012a
46.	*Z.wui*	CIB 097690	Hubei, China	JN688880	Li et al. 2012a
47.	*Z.yaoshanensis*	NHMG150408	Guangxi, China	MG322122	Chen et al. 2018
48.	*Z.yaoshanensis*	NHMG150404	Guangxi, China	MG322121	Chen et al. 2018
49.	*Z.yinggelingensis*	HN2018002	Hainan, China	MW192130	Genbank
50.	*Z.yunnanensis*	Rao3494	Longling, Yunnan, China	JX219429	Li et al. 2012b
51.	*Z.yunnanensis*	Rao3496	Longling, Yunnan, China	JX219428	Li et al. 2012b
52.	*Z.zhoukaiyae*	AHURhaDb-150420-03	Anhui, China	KU601499	Pan et al. 2017
53.	*Z.zhoukaiyae*	AHURhaDb-150420-01	Anhui, China	KU601500	Pan et al. 2017
54.	*Zhangixalusthaoae* sp. nov.	ROM38011	Lao Cai, Vietnam	JX219427	Li et al. 2012b
55.	*Zhangixalusthaoae* sp. nov.	IEBR A 5136	Lao Cai, Vietnam	LC762092	This study
56.	*Zhangixalusthaoae* sp. nov.	IEBR A 5137	Lao Cai, Vietnam	LC762093	This study
57.	*Zhangixalusthaoae* sp. nov.	IEBR A 5138	Lao Cai, Vietnam	LC762094	This study

### ﻿Molecular data and phylogenetic analyses

We used the protocols of Kuraishi et al. (2013), modified by Nguyen et al. (2015a), for DNA extraction, amplification, and sequencing. Fragments of the 16S rRNA mitochondrial DNA gene were amplified using the same primers used by Kuraishi et al. (2013).

For the phylogenetic analyses, 55 sequences of 26 *Zhangixalus* species were combined with two sequences of *Rhacophoruskio* as outgroups (Table 1).

Chromas Pro software (Technelysium Pty Ltd, Tewantin, Australia) was used to edit the sequences, which were aligned using MAFFT v. 7 (Katoh and Standley 2013) with default settings. We then checked the initial alignments by eye and adjusted them slightly. Phylogenetic trees were constructed using IQ-TREE v. 1.6.12 (Nguyen et al. 2015b) while maximum-likelihood bootstrap support (MLBS) was evaluated by ultrafast bootstrap approximation with 1000 replicates (Hoang et al. 2018) (ML). Prior to Bayesian analyses, we chose the optimum substitution models for entire sequences by using ModelFinder implemented in IQ-TREE based on the Bayesian information criterion (BIC) (Kalyaanamoorthy et al. 2017). According to ModelFinder, the best-fit model for ML analysis was TIM2+F+I+G4. Because the TIM2 model and F parameter are not implemented in MrBayes, we selected the next best-fit model for our Bayesian-inference (BI) analysis, which was the general time reversible model (GTR; Tavaré 1986) with a proportion of invariable sites and a gamma shape parameter (Alpha 0.215). The BI phylogenetic construction was done in MrBayes v. 3.2.7a (Ronquist et al. 2012) with two independent runs of four Markov Chains for 10,000,000 generations. A tree was sampled every 100 generation, and a consensus topology was calculated for 75,001 trees after discarding the first 25,000 trees using the relative burn-in option (25% of trees discarded). We checked parameter estimates and convergence using TRACER v. 1.5 (Rambaut and Drummond 2009). We regarded tree nodes in the ML tree with bootstrap values of 95% or greater as sufficiently resolved (Hoang et al. 2018), and nodes with a BPP of 95% or greater as significant in the BI analysis (Leaché and Reeder 2002).

### ﻿Morphological characters

A total of 34 measurements were taken with digital calipers to the nearest 0.1 mm (Nguyen et al. 2016). Abbreviations are as follows: **SVL**: snout–vent length, **HW**: head width (across angle of jaws), **HL**: head length (from back of mandible to tip of snout), **MND**: distance from jaw angle to nostril, **MFED**: distance from back of mandible to front of the eye, **MBED**: distance from back of mandible to back of the eye, **SNL**: snout length (from anterior corner of eye to tip of snout), **ED**: eye diameter, **UEW**: maximum width of upper eyelid, **IND**: internarial distance, **IOD**: interorbital distance (minimal distance between orbits), **AED**: distance between anterior corners of eyes, **PED**: distance between posterior corners of eyes, **NS**: distance from nostril to tip of snout, **EN**: distance from anterior corner of eye to nostril, **TYD**: maximal tympanum diameter, **TED**: distance from anterior margin of tympanum to posterior corner of eye, **Ua**: upper arm length (from axilla to elbow), **Fa**: lower arm and hand length (from elbow to tip of third finger), **NPL**: nuptial pad length, **F1–4**: length of fingers I–IV (from basis of finger to tip of finger), **FeL**: thigh length from vent to knee, **TbL**: tibia length from knee to tarsus, **TbW**: maximal tibia width, **FL**: foot length from tibiotarsal joint to tip of fourth toe, **T1–5**: length of toes I–V, **IML**: inner metatarsal tubercle length. Terminology for describing the webbing formula followed Glaw and Vences (2007). Sex was determined by the presence of nuptial pads and gonadal inspection.

## ﻿Results

### ﻿Phylogenetic analyses

**Figure 1.**
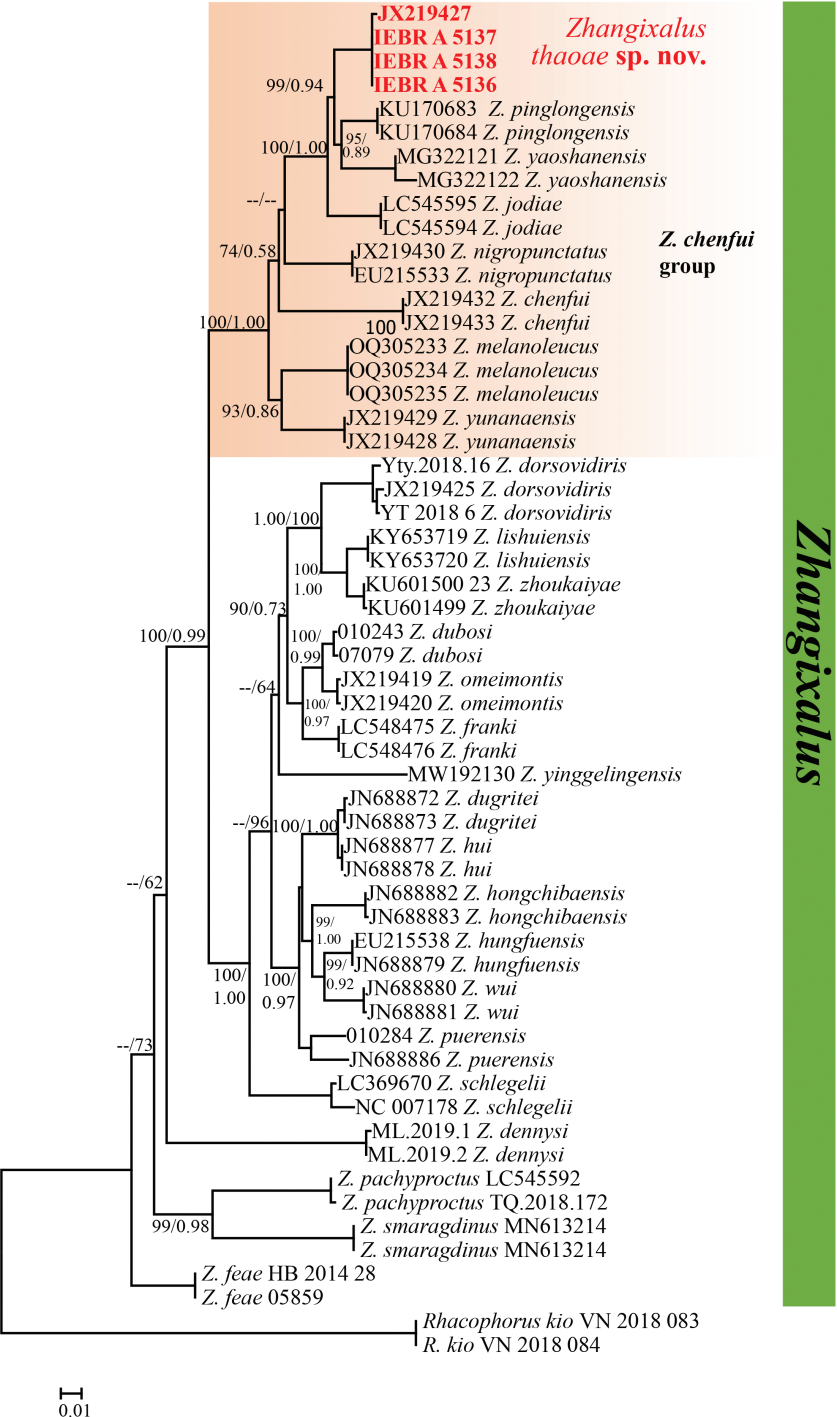
BI tree from a 1033 bp sequence of mitochondrial 16S rRNA gene of *Zhangixalus* and outgroup species. Numbers above and below branches are Bayesian posterior probabilities (BPP) and ML bootstrap. For GenBank accession numbers, refer to Table 1.

The aligned 16S sequences yielded a total of 1,033 characters. Of 1033 nucleotide sites, 309 were variable and 295 were parsimony informative within the analysed *Zhangixalus* species. Nucleotide frequencies were A = 37.7%, T = 24.4%, C = 20.7%, and G = 17.2% (data for ingroup only). Our phylogenetic analyses employing ML and BI methods yielded identical topologies, and only the BI tree is presented in Fig. 1.

Phylogenetically, the undescribed species of *Zhangixalus* from Y Ty Commune, Bat Xat District, Lao Cai Province, Vietnam was clustered with seven species in the *Z.chenfui* group with a well-supported node (both 100% in the ML and BI analyses). Furthermore, the undescribed *Zhangixalus* species was found to be most closely related to a clade consisting of *Z.pinglongensis* and *Z.yaoshanensis*, with significantly high support value in the BI analysis (99%) and a high support value from ML analysis (94%). The genetic distance among the examined sequences ranges from 3.23% (between *Zhangixalus* sp. from Lao Cai Province and *Z.pinglongensis*) to 8.10% (between *Z.chenfui* and *Z.yaoshanensis*) (Table 2).

**Table 2.** Uncorrected pairwise distances (*p*-distance) among *Zhangixalus* species analysed.

	1.	2.	3.	4.	5.	6.	7.	8.	9.	10.	11.	12.	13.	14.	15.	16.	17.	18.	19.	20.	21.	22.	23.	24.	25.	26.
1. *Zhangixalusthaoae* sp. nov.	0.00–0.19																									
2. *Z.chenfui*	7.42	0.00																								
3. *Z.dennysi*	10.72	11.12–11.32	0.19																							
4. *Z.dorsoviridis*	8.59–8.69	9.29–9.38	9.76–40.15	0.39–0.78																						
5. *Z.duboisi*	7.62–7.91	8.69–8.89	9.07–9.27	4.88–5.27	0.29																					
6. *Z.dugritei*	8.02–8.99	8.22–8.32	9.86–10.16	6.05–6.54	4.40–4.59	0.11																				
7. *Z.feae*	8.81–8.99	9.00–9.09	8.30–8.50	9.00–9.38	8.31–8.40	8.02–8.21	0.00																			
8. *Z.franki*	7.81–7.97	8.50–8.70	9.07–9.51	5.18–569	2.73–2.90	4.88–5.18	8.21–8.70	0.00																		
9. *Z.hongchibaensis*	8.71–8.90	9.21–9.30	10.95–11.24	6.55–7.04	5.09–5.38	3.62–3.81	8.72–8.90	5.57–5.91	0.10																	
10. *Z.hui*	8.21–8.31	8.22	9.86–10.06	6.35–6.54	4.50–4.59	0.49–0.59	8.12–8.21	4.79–4.87	3.91–4.01	0.00																
11. *Z.hungfuensis*	8.62–8.72	9.21	10.08–10.27	5.97–6.16	5.09–5.19	3.71–3.81	8.81–8.90	5.19–5.50	3.52–3.62	3.62	0.00															
12. *Z.jodiae*	3.90	7.32	10.32–10.52	8.98–9.27	7.41–7.61	7.81–7.91	8.31–8.40	7.71–7.76	8.90–8.99	8.01	8.90	0.00														
13. *Z.lishuiensis*	9.09–9.19	8.61	9.47	4.00–4.20	4.50–4.59	5.87–5.96	7.93–8.02	4.69–4.97	6.56–6.65	5.87	5.48	9.08	0.00													
14. *Z.melanoleucus*	6.43	7.22	10.81–11.00	8.29–8.68	8.20–8.39	7.71–7.81	8.80–8.89	7.61–7.86	8.70–8.80	7.91	8.71	7.21	8.89	0.00												
15. *Z.nigropunctatus*	5.56–5.76	6.93	10.33–10.53	9.08–9.28	8.11–8.30	8.11–8.20	8.41–8.50	7.71–7.96	9.29–9.38	8.30	9.00	5.65	8.90	5.46	0.00											
16. *Z.omeimontis*	7.91–8.11	8.79–8.89	9.17–9.56	4.98–5.18	1.27–1.37	4.50–4.59	8.21–8.30	3.03–3.11	5.38–5.58	4.40	4.99–5.09	7.32	4.40–4.59	8.19–8.59	8.30	0.20										
17. *Z.pachyproctus*	10.54–10.83	10.94–11.13	10.04	9.76–10.34	9.46–9.66	9.96–10.25	7.62–7.82	9.76–10.13	10.36–10.65	9.86–10.06	10.18	10.82–11.01	9.28–9.47	10.92–11.11	10.44–10.63	9.37–9.56	0.19									
18. *Z.pinglongensis*	3.23–3.33	7.24	10.36	8.80–8.90	7.72–7.82	8.02–8.12	8.62–8.71	7.83–7.88	8.81–8.91	8.22	8.42	4.01	8.61	6.74	5.68	7.82–7.92	10.65–10.85	0.00								
19. *Z.puerensis*	7.92–8.71	8.22–8.62	9.29–9.77	6.35–6.74	4.89–5.28	3.42–3.61	7.73–8.12	5.08–5.59	3.82–4.79	3.32–3.42	3.62–3.82	7.81–8.21	5.68–6.16	8.21–8.30	8.69–8.90	4.79–4.89	9.58–9.86	7.93–8.13	2.83							
20. *Z.schlegelii*	8.50–9.20	9.00–10.11	10.25–10.65	7.32–7.43	5.87–6.16	6.05–6.65	7.92–8.32	5.96–6.53	6.16–6.56	5.57–6.06	6.07–6.17	8.69–9.38	6.84–7.14	8.20–8.40	8.89–9.38	5.87–6.26	9.28–9.58	9.00–9.40	5.86–6.36	1.17						
21. *Z.smaragdinus*	10.73–10.83	10.94	10.33	10.93–11.32	10.54	10.74–10.84	8.79–8.80	10.63–11.07	11.44–11.53	10.84	11.06	10.43	10.35	10.82	10.54	10.44	8.38	10.75	10.36–10.45	9.96–10.17	0.00					
22. *Z.wui*	8.61–8.81	9.01–9.11	9.87–10.17	6.65–6.94	4.99–5.09	3.52–3.71	8.91–9.10	5.18–5.49	3.91–4.10	3.23–3.32	2.84–2.94	8.90–8.99	6.56–6.65	8.41–8.50	8.99–9.09	4.99–5.09	10.39–10.65	8.91–9.01	4.11–4.30	6.26–6.56	11.53–11.63	0.10				
23. *Z.yaoshanensis*	3.80–4.68	7.62–8.10	10.53–11.10	9.07–9.85	7.90–8.68	8.50–9.28	9.29–9.77	7.90–8.79	9.68–10.36	8.69–9.38	9.30–9.88	4.68–5.36	9.18–9.67	6.82–7.79	5.95–6.53	8.29–8.88	11.21–11.89	3.62–4.30	8.69–9.58	9.18–9.97	10.82–11.40	9.38–10.07	0.88			
24. *Z.yinggelingensis*	8.81–8.91	8.42	10.76–10.86	7.34–7.44	6.17	5.97–6.07	8.54–8.63	6.37–6.54	7.90–8.68	6.07	6.67	9.20	7.05	8.71	8.52	6.07–6.17	10.37–10.57	8.43	6.08–6.17	7.84–8.83	11.06	7.06	8.81–9.10			
25. *Z.yunnanensis*	6.54	6.93	10.23–10.43	8.40–8.50	8.49–8.68	7.82–7.92	8.20–8.21	8.01–8.39	8.90–9.00	7.82	8.71	6.43	8.50	4.97	5.46	8.20–8.29	10.73–10.93	6.74	8.31–8.41	8.31–8.61	9.85	8.71–8.81	6.83–7.41	8.52		
26. *Z.zhoukaiyae*	8.61–8.81	8.52–8.62	9.68–9.78	3.81–4.01	4.40–4.60	5.68–5.87	8.04–8.23	4.79–5.19	6.56–6.76	5.58–5.68	5.39–5.49	8.41–8.50	1.56–1.66	8.60–8.70	8.61–8.71	4.21–4.50	8.90–9.19	8.42–8.52	5.68–6.07	6.65–6.86	9.68–9.78	6.56–6.66	8.90–9.48	6.56–6.66	8.32–8.41	0.10

### ﻿Taxonomic account

#### ﻿ Zhangixalus thaoae sp. nov.

Taxon classificationAnimaliaAnuraRhacophoridae

69706455-064C-5527-8D62-BEC5AD7D25A2

https://zoobank.org/DB9E5E8E-3E66-4AB1-B837-8FB8D485E982

Fig. 2

##### Material examined.

***Holotype***: Vietnam • ♂; Y Ty Commune, Bat Xat District, Lao Cai Province, Northwestern Vietnam; 22°37'17.6"N, 103°37'23.5"E; 1883 m a.s.l.; 01 May 2019; C. V. Hoang and A. M. Luong leg.; IEBR A 5136; GenBank: LC762092.1. ***Paratypes***: Vietnam • 2 ♂; same locality as for holotype; same geo-coordinates; same altitude; same collection date; same collectors; IEBR A 5137, 5138; GenBank: LC762093.1, LC762094.1.

##### Diagnosis.

**Figure 2.**
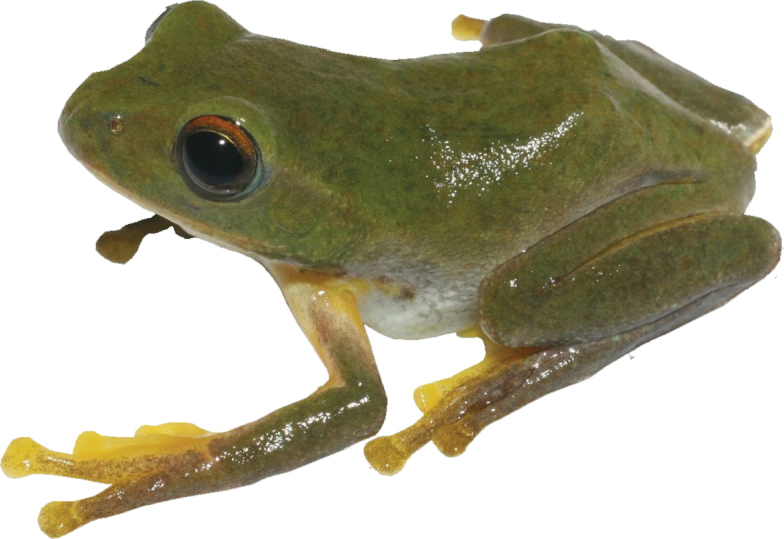
Adult male holotype (IEBR A 5136) of *Zhangixalusthaoae* sp. nov., in life, from Y Ty Commune, Bat Xat District, Lao Cai Province, northwestern Vietnam.

The new species is placed in the genus *Zhangixalus* based on some morphological characters: dorsum green and smooth; body size medium (SVL 30.1–32.2 in males); fingers webbed; tips of digits expanded into large disks, bearing circum-marginal grooves; absence of dermal folds along limbs; absence of supracloacal fold and tarsal projection (Fig. 2).

The new species is distinguished from its congeners by a combination of the following characteristics: 1) dorsal surface of head and body green without spots; 2) axilla and groin cream with a black blotch; 3) ventrum cream-colored without spots; 4) chin cream, with grey marbling; anterior part of thigh and ventral surface of tibia orange, without blotch; posterior parts of thigh orange with a large, black blotch; 5) ventral side of webbing orange, with some grey; 6) iris red-bronze, pupils black. 7) finger webbing formula I1¼-1¼II1-2III1-1IV, and toe webbing formula I½-½II0-1½III¼ -1¾IV1¾-½V.

##### Description of the holotype

**(male).** Size medium (SVL 32.2 mm), body robust; head slightly compressed, head length nearly equal to the width (HW 12.1 mm, HL 11.7 mm), convex above; snout round, slightly protruding beyond lower jaw in lateral view, and longer than the horizontal diameter of eye (SNL 5.4 mm, ED 4.2 mm); canthus rostralis round, loreal region oblique, concave; interorbital distance wider than internarial distance and upper eyelid (IOD 4.3 mm, IND 3.1 mm, UEW 3.0 mm); distance between anterior corners of eyes about 69.30% of the distance between posterior corners of eyes; nostrils round, without lateral flap of skin, closer to tip of snout than to eye; pupil oval, horizontal; tympanum distinct, round, about half the size of eye diameter, and twice greater than distance between tympanum and eye (TYD 2.1, TYE 1.0); pineal ocellus and spinules on upper eyelid absent; vomerine teeth well developed, in two oblique ridges; choanae round; tongue deeply notched posteriorly; supratympanic fold weakly.

***Forelimbs*** robust, upper arm short, nearly one-half of hand length (Ua 6.2 mm, Fa 14.6 mm), dermal fringe along the outer edge of forearm absent; relative finger lengths I<II<V<III; tips of fingers with enlarged discs with distinct circum-marginal grooves; disc of finger III approximately 1.5 times of the width of finger III (fd3/fw3 1.5), greater than tympanum diameter (fd3/TYD 1.27); webbing formula I1¼-1¼II1-2III1-1IV, subarticular tubercles distinct, blunt, round, formula 1, 1, 2, 2; nuptial pads prominent, oval, smooth.

***Hindlimbs*** long and thin, heels overlapping when held at right angles to the body; tibia length about four times greater than tibia width (TbL 13.9 mm, TbW 3.1 mm), longer than thigh length (FeL 13.3 mm), shorter than foot length (FL 19.0 mm); relative toe lengths I<II<III<V<IV; tips of toes with enlarged discs with distinct circum-marginal grooves, discs slightly smaller than those of fingers; webbing formula I½-½II0-1½III¼-1¾IV1¾-½V; subarticular tubercles distinct, blunt, round, formula 1, 1, 2, 3, 2; inner metatarsal tubercle small (IML 1.6 mm); dermal ridge along the outer edge of tibia and tarsus absent; dermal projection at tibiotarsal articulation absent.

***Skin texture***: dorsal surface of head and body smooth; supratympanic fold weakly developed, throat and chest smooth, belly rough; ventral surface of fore- and hindlimbs smooth.

***Coloration in life***: iris red-bronze, pupil black; dorsal surface of head and body green without spots; dorsal surface of fore and hind limbs green, upper side of fingers II and II and toes I, II, and III yellow, all tip of fingers and toes yellow; axilla cream and groin cream with a black blotch; anterior part of thigh and ventral surface of tibia orange without spots; posterior parts of thigh orange with a large black blotch; lower jaw cream, with grey marbling, and throat region white; ventral side of webbing orange with some gray pattern, nuptial pads grey.

***Coloration in preservative***: As in life, but with green dorsal surface fading to dark blue; and ventral side of body, limbs, and upper side of fingers I and II, upper side of toes I, II, and III fading to light yellow.

##### Variation.

**Figure 3.**
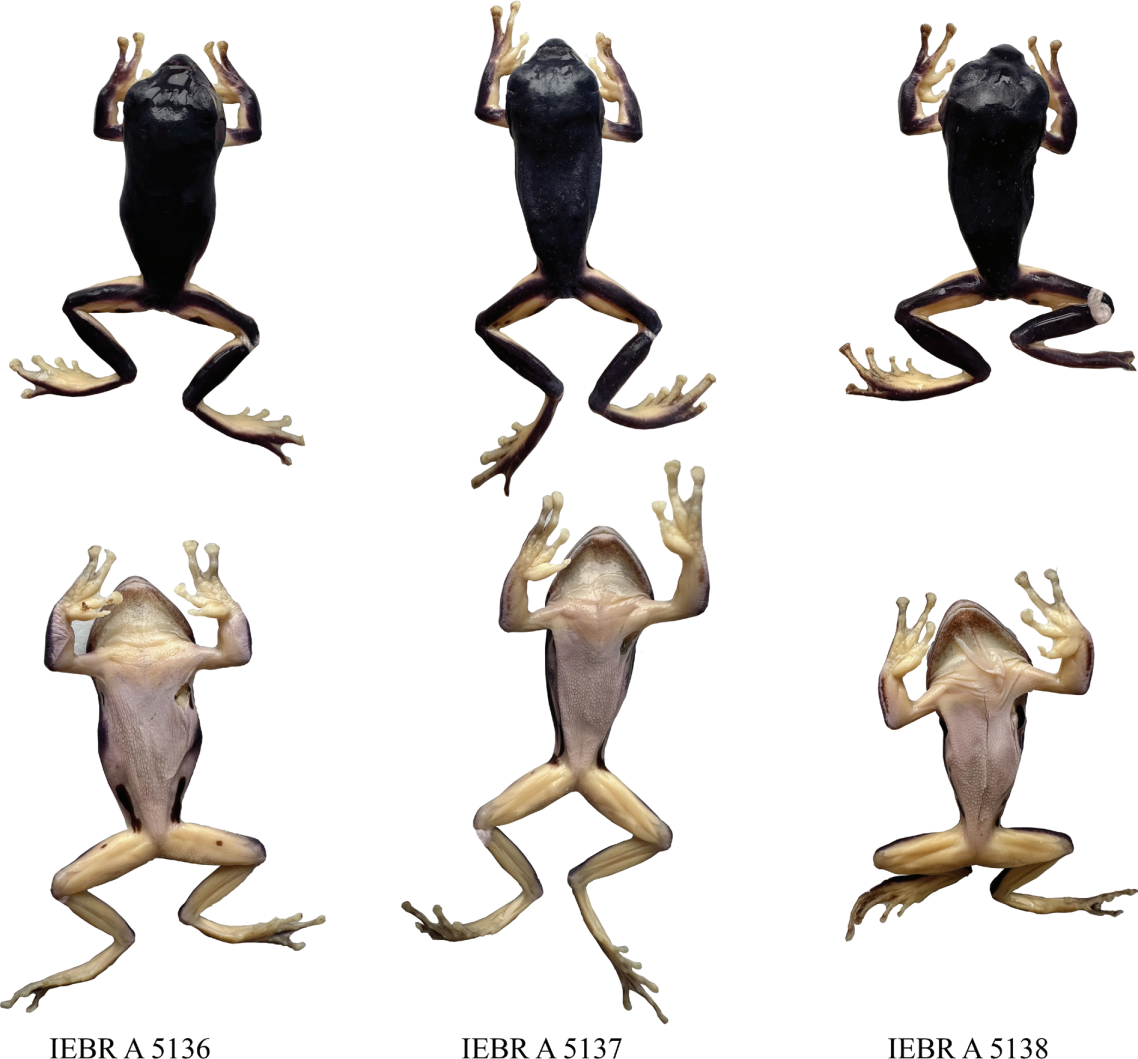
The variation of morphological characteristic of *Zhangixalusthaoae* sp. nov. IEBR A 5136 is the holotype, and the two remaining samples are paratypes.

Ground color of dorsum light green; ventral surface cream, without spots. The size of blotches in the axilla, groin, and posterior thigh region of the paratype is smaller than in the holotype (Fig. 3). For measurements of the type series, see Table 3.

**Table 3.** Measurements (in mm) of *Zhangixalusthaoae* sp. nov.

Number	IEBR A 5136	IEBR A 5137	IEBR A 5138
**Sex**	Male (holotype)	Male	Male
**SVL**	32.2	32.0	30.1
**HW**	12.1	11.7	12.8
**HL**	11.7	11.3	13.1
**MND**	9.5	9.8	10.8
**MFED**	7.5	7.2	10.6
**MBED**	3.8	4.5	4.1
**SNL**	5.4	5.1	5.4
**ED**	4.2	3.4	3.9
**UEW**	3.0	3.0	3.5
**IND**	3.1	3.9	4.0
**IOD**	4.3	4.6	4.7
**AED**	7.0	7.1	7.5
**PED**	10.1	10.4	11.5
**NS**	3.1	2.8	3.2
**EN**	2.9	2.6	3.2
**TYD**	2.1	2.0	2.5
**TED**	1.0	1.2	1.4
**Ua**	6.2	6.1	7.0
**Fa**	14.6	16.1	16.6
**F1**	3.0	4.0	3.1
**F2**	4.2	5.2	5.4
**F3**	6.1	6.4	7.2
**F4**	4.8	5.7	5.3
**FeL**	13.3	13.2	14.0
**TbL**	13.9	13.9	14.0
**TbW**	3.4	3.1	3.3
**FL**	19.0	20.3	21.8
**T1**	3.6	5.0	4.5
**T2**	6.2	7.4	6.8
**T3**	9.2	10.3	10.7
**T4**	11.8	13.5	13.6
**T5**	10.0	10.9	11.3
**IML**	1.6	1.8	1.7
**HW/HL**	1.03	1.04	0.97
**SNL/HW**	0.44	0.43	0.43
**NS/EN**	1.08	1.07	0.99
**ED/SNL**	0.78	0.66	0.72
**TYE/TYD**	0.46	0.62	0.57
**TYD/ED**	0.50	0.59	0.64
**HAL/FLL**	2.36	2.64	2.37
**TBL/TL**	1.05	1.05	1.01

##### Etymology.

The species is named after the first author’s wife, Nguyen Thi Thanh Thao, as a token of gratitude for her understanding and strong support of his research activity. We recommend Thao’s Tree Frog as the English common name and Ếch cây thảo as the Vietnamese common name.

##### Male secondary sexual characters.

Male specimens with nuptial pad present on base of the finger I and external single subgular vocal sac.

##### Natural history notes.

**Figure 4.**
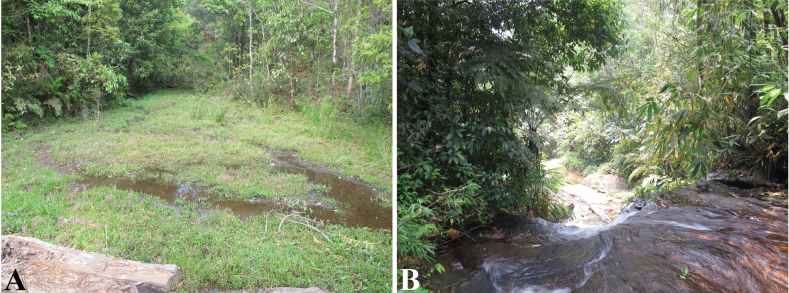
Habitat of the new species in Y Ty Commune, Bat Xat District, Lao Cai Province **A** collection site with a small puddle **B** the stream near the biotope of the holotype.

Specimens were collected between 19:00 and 24:00h on a branch about 1 m above the ground. The ground consisted of mountain soil and puddles, and there was a small stream about 2 m away (Fig. 4A). The habitat was an undisturbed evergreen forest on a granite mountain (Fig. 4B). Other tree frogs that were found at the site were *Polypedates* sp., of the *P.leucomystax* species complex; *Zhangixalusduboisi* (Ohler, Marquis, Swan & Grosjean, 2000); *Hylaannectans* (Jerdon, 1870); and *Gracixalusgracilipes* (Bourret, 1937). Females, larval stages, and eggs of the new species are unknown.

##### Distribution.

**Figure 5.**
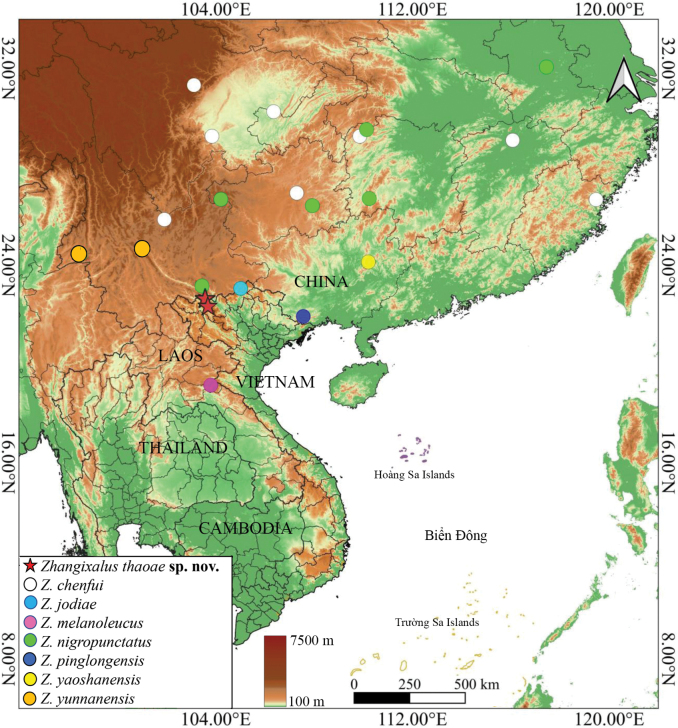
The distribution of species of the *Zhangixaluschenfui* group.

*Zhangixalusthaoae* sp. nov. is currently known only from the type locality (Fig. 5). The species was recorded at an elevation of approximately 1,880 m a.s.l.

##### Conservation status.

The new species is expected to be found in the evergreen forest of Guangxi Province, southern China, because the terrain there consists mostly of granite mountains, but in Yunnan Province, China, which contains mostly limestone terrain, the species is not expected. However, the geographic distribution of the species needs to be confirmed by further studies. Because there is a lack of information on the species’ abundance and distribution, we suggest that it be considered as Data Deficient following IUCN Red List categories (IUCN 2023).

##### Comparisons.

We compare *Zhangixalusthaoae* sp. nov. with other species of *Zhangixalus* occurring in Vietnam and elsewhere.

The new species mostly resembles *Z.yaoshanensis* by the combination of the following characteristics: head as long as wide in *Zhangixalusthaoae* sp. nov. (HL/HW 0.96–1.02), posterior parts of thigh orange with a large black blotch, ventral surface of tibia orange, iris red-bronze with black pupil in *Zhangixalusthaoae* sp. nov. vs head wider than long (HL/HW = 0.83); posterior thigh surface and ventral surface of tibia red-orange without spot, iris pale yellowish gold with a network of fine dark gold reticulations in *Z.yaoshanensis* (Chen et al. 2018).

*Zhangixalusthaoae* sp. nov. can be distinguished from other species in the genus *Zhangixalus* by its smaller size (SVL 30.1–32.2 mm) vs SVL >50 mm in the following species: 47.0–70.5 (Wilkinson and Rao 2004; Ohler 2009; Jiang et al. 2016) in *Z.burmanus*; 68–92 mm (Fei et al. 2010) in *Z.dennysi*; 53.1–67.2 mm (Ohler et al. 2000; Orlov et al. 2001; Ziegler et al. 2014) in *Z.duboisi*; 68–116 mm (Fei et al. 2010) in *Z.feae* (Boulenger, 1893); 77.9–85.8 mm (Ninh et al. 2020) in *Z.franki*; 52–66 mm (Fei et al. 2010), 52–65 mm (Liu 1950) in *Z.omeimontis*; 74.2 mm (Luu and Calame 2014), 73.4–78.2 mm in *Z.pachyproctus* (Yu et al. 2019); and 76.3–79.6 mm in *Z.smaragdinus* (Yu et al. 2019).

*Zhangixalusthaoae* sp. nov. can be distinguished from other *Zhangixalus* species of a similar size in having a different coloration pattern: dorsum green without blotches or spots in the new species vs light or dark green with many white or brown spots or blotches in various sizes in *Z.dugritei* (David 1872; Li et al. 2012a), *Z.hongchibaensis* (Li et al. 2012a), *Z.hui* (Liu 1945), and *Z.wui* (Li et al. 2012a).

*Zhangixalusarboreus* has a green dorsum with numerous dark spots (Okada and Kawano 1924), which is absent in *Zhangixalusthaoae* sp. nov.

*Zhangixalusachantharrhena* has a green dorsum, without spots, and a cream venter, without the brown flecks of the new species (Harvey et al. 2002).

*Zhangixalusarvalis* has a white line along flanks, which is absent in *Z.thaoae* sp. nov. (Lue et al. 1995).

*Zhangixaluschenfui* has a brown ventrum, with a dark-brown pattern, not cream-colored, without a dark-brown pattern, as in *Zhangixalusthaoae* sp. nov. (Liu 1945).

**Figure 6.**
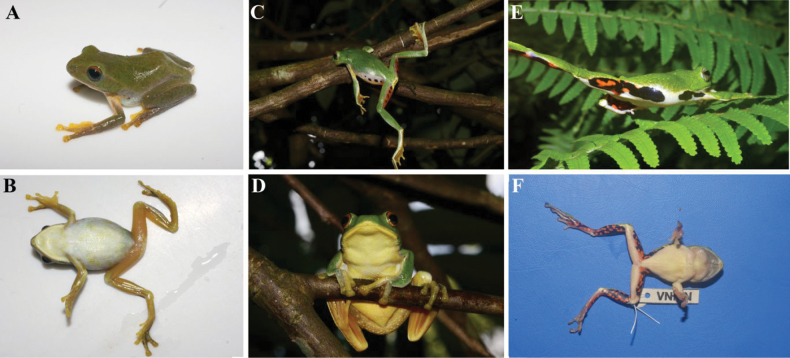
Dorsal and ventral views of three *Zhangixalus* species in life (except for F, which was immediately photographed after the specimen was anesthetized) **A, B***Zhangixalusthaoae* sp. nov. IEBR.A 5136 **C, D***Z.dorsoviridis* VNMN 06156 **E, F***Z.jodiae* VNMN 07122.

**Figure 7.**
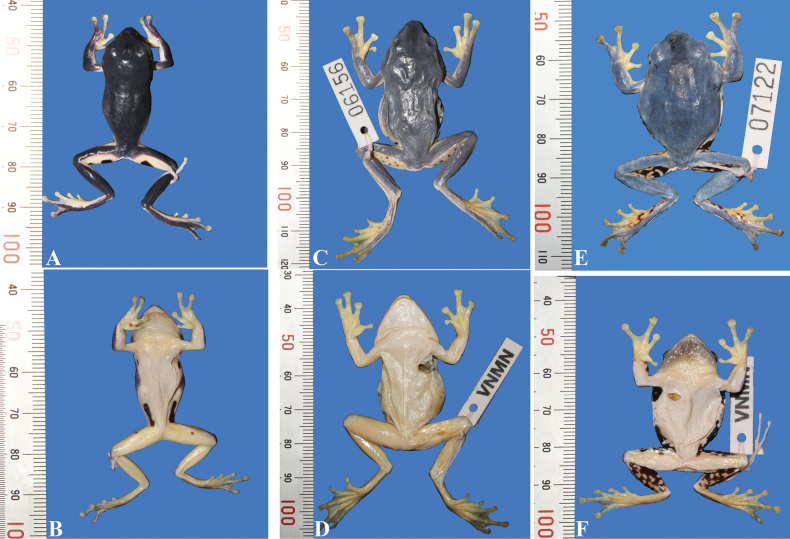
Dorsal and ventral views of three *Zhangixalus* species in preservative **A, B***Zhangixalusthaoae* sp. nov IEBR.A 5136 **C, D***Z.dorsoviridis* VNMN 06156 **E, F***Z.jodiae* VNMN 07122.

*Zhangixalusdorsoviridis* has cream flanks with many black blotches and the anterior thigh is orange with some irregularly sized black circles (Bourret 1937) (Figs 6C, D, 7C, D). In *Z.thaoae* sp. nov., the flanks are cream with one single black spot and the posterior thigh is orange with a single large black blotch (Figs 6A, B, 7A, B).

*Zhangixalusdulitensis* is pea-green dorsally with some white dots, the head and back have purplish dots, there is purplish line from eye to eye around the snout and passing through the nostrils, and there is reddish-brown patch on each eyelid (Boulenger 1892; Haas et al. 2012). *Zhangixalusthaoae* sp. nov. does not have this color pattern.

*Zhangixalusjarujini* has a reddish-brown dorsum with irregular dark-brown markings, while *Z.thaoae* sp. nov. has a green dorsum without any markings.

*Zhangixalusjodiae* has black and orange blotches interposed on anterior, posterior part of thighs and ventral surface of tibia (Figs 6E, F, 7E, F); *Z.thaoae* sp. nov. does not have this color pattern, but rather a large, black blotch on an orange background (Figs 6A, B, 7A, B).

*Zhangixalusleucofasciatus* exhibits a cream axilla without dark spots, in contrast to a black blotch on the cream axilla of *Z.thaoae* sp. nov.; additionally, *Z.leucofasciatus* possesses a white stripe along the upper lip, body and limbs, a feature that is absent in *Z.thaoae* sp. nov. (Liu and Hu 1962; Fei et al. 2010).

*Zhangixaluspinglongensis* has flanks, anterior and posterior surfaces of the thigh covered with black blotches and white spots (Mo et al. 2016). In contrast, *Z.thaoae* sp. nov. lacks similar black blotches with white spots on its flanks, anterior and posterior surfaces of the thighs.

*Zhangixalusminimus* is characterized by a dark-brown mottling pattern on its hands and feet, which is absent in *Z.thaoae* sp. nov. (Rao et al. 2006).

*Zhangixalusmelanoleucus* is whitish cream with an irregular black pattern on the ventral surface of its thighs, shanks, dorsal surfaces of the feet and fingers I, II, and III (Brakels et al. 2023). Meanwhile, in *Z.thaoae* sp. nov., the ventral surface of the thighs, shanks, dorsal surfaces of the feet and fingers I, II, and III are orange, without any irregular black pattern.

*Zhangixalusmoltrechti* has a red-orange anterior and posterior thigh with multiple dark spots, while the thigh in *Z.thaoae* sp. nov. has an orange anterior with no spots and an orange posterior with a single large blotch (Boulenger 1908; Fei et al. 2010). Furthermore, the webbings of the foot in *Z.moltrechti* are red-orange, with dark spots, while those in *Z.thaoae* sp. nov. are yellow with no spots.

*Zhangixalusnigropunctatus* has yellow flanks and posterior thigh with some black blotches, in contrast to *Z.thaoae* sp. nov., in which the flank is cream-colored and the posterior thigh is orange, both featuring a single black spot (Liu et al. 1962; Fei et al. 2010). In *Z.nigropunctatus*, there is also a white stripe along the flanks and limbs, separating the dorsal and ventral sides, which is a feature that is absent in *Z.thaoae* sp. nov.

*Zhangixalusschlegelii* has flanks and groin without spots, while in *Z.thaoae* sp. nov. these each have a large black blotch (Günther 1858). A white stripe along the flanks and limbs, separating the dorsal and ventral sides, is present in *Z.schlegelii* but absent in *Z.thaoae* sp. nov. Furthermore, there is a prominent supratympanic fold in *Z.schlegelii*, which is only weakly visible in *Z.thaoae* sp. nov.

*Zhangixalusyinggelingensis* has green dorsal head, body and limbs, adorned with a small number of very fine white spots (Chou et al. 2007), which are absent in *Z.thaoae* sp. nov. In addition, the supratympanic fold in *Z.yinggelingensis* is prominent, whereas it is weakly visible in *Z.thaoae* sp. nov.

*Zhangixalusyunnanensis* has greyish webbings and yellowish-brown iris, which are respectively orange and red-bronze in *Z.thaoae* sp. nov. (Pan et al. 2024). Furthermore, *Z.yunnanensis* exhibits a somewhat broader head (IND/IOD 0.96-1), which is proportionally smaller relative to the body (HL/SVL 0.31-0.33) compared to the head of *Z.thaoae* sp. nov. (IND/IOD 0.72-0.85, HL/SVL 0.37-0.43).

*Zhangixaluszhoukaiyae* has yellowish posterior thigh with irregular greyish blotching, whereas the posterior thigh of *Z.thaoae* sp. nov. is orange without spots. In *Z.zhoukaiyae*, the pupil is dark charcoal-grey and the iris is golden-yellow, while in *Z.thaoae* sp. nov. the pupil is black, and the iris is red-bronze (Pan et al. 2017).

A more detailed comparison of morphological differences between *Z.thaoae* and other members in the *Z.chenfui* group can be found in Table 4.

**Table 4.** Comparison of morphology character of species within *Z.chenfui* group.

Species	SVL	Dorsum color	Vomerine teeth	Ventral color	Flank coloration	Snout shape	The color pattern of the thigh	Fingers web formula	Toes web formula	Source
*Zhangixalusthaoae* sp nov.	30-32.2 (M)	Smooth, and green without spots	Present	Lower jaw cream with grey and throat region white belly cream without spots	Flank cream with a black blotch	Rounded	The anterior part of the thigh is orange without spots; the posterior part of the thigh is orange with a large black blotch	I1¼-1¼II1-2III1-1IV	I½-½II0-1½III¼ -1¾IV1¾-½V	This study
*Z.chenfui*	33-41 (M) 48-55 (F)	Skin with granules above	Present	Ventral brown with dark-brown pattern	Flanks orange with blotches, have a black strip along flank isolated upper and lower part of the body	Rounded	Anterior of thigh orange with blotches	–	–	Liu et al. 1945; Fei et al. 2009
*Z.jodiae*	36.1–39.8 (M)	Dorsal surface of the head and body green without spots	Present	Lower jaw region greyish, chest and belly cream	Flank cream, axilla, and groin with large black blotches	Rounded	The dorsal surface is green without spots, the front-rear parts of the thigh, and the ventral surface of the tibia black with orange blotches	I1-1II1-1III2-1IV	I1-1II½-1III0-1½IV1-½V	Nguyen et al. 2021
*Z.melanoleucus*	34.4-36.3 (M) 53.7 (F)	Dorsum smooth and uniform green with several dark and light-green spots	Present	Throat grey with dark grey margins, chest and belly immaculate white	Flank white, covered by an irregular black pattern, groin cream	Rounded	The anterior and posterior surfaces of the thighs are white cream covered with an irregular black pattern, ventral surfaces of the thighs cream	I2½-3II2-3 III2¼- 2IV	I2-2½II1-2III1-2 IV2-1V	Brakels et al. 2023
*Z.nigropunctatus*	32.0–37.0 (M) 44-45(F)	Dorsum smooth and green	_	Lower jaw grey, chest and belly white	Flanks orange with blotches has a white strip along isolated dorsal and ventral side	Rounded	Dorsal surface green, anterior and posterior surfaces of thighs orange and has some black blotches on posterior of thigh	–	–	Fei et al. 2009
*Z.pinglongensis*	32.0–38.5 (M)	Dorsum smooth and green	Present	Lower jaw grey, chest and belly cream	Flank covered with black blotches with white spots or white spots with a faint orange tint	Rounded	The anterior and posterior surfaces of the thigh covered with black blotches with white spots or white spots with a faint orange tint	I 1-–1-II 1^+^–1^+^III2-–2-IV	I1^+^–1-II 2^+^–2-III 2^+^–3-IV 3-–2^+^V	Mo et al. 2016
*Z.yaoshanensis*	31.6–36.4 (M) 51.1 (F)	The dorsal surface is smooth and green, with or without faint spots,	Present	Throat grey in males, ventral green without spots	The posterior surface of the flanks is orange-red without spots	Point	Dorsal surface green with or without faint green spots, anterior and posterior surface of thighs orange-red without spots	I1-–1-II1–1- III1^+^–2-IV	I 1^+^–1-II1^+^–1^+^III 2^+^–2 IV2^+^–2^+^V	Chen et al. 2018
*Z.yunnanensis*	31.3-36.0 (M) 47.6-48.6 (F)	Smooth and green	Present	Throat black	Cream mottled with greyish brown	Rounded	Black blotches in axilla, groin, and posterior part of thigh	I1–1II1–2III1–1IV	I1–1II0.5–2.5III1–2IV2–0.5IV	Pan et al. 2024

## ﻿Discussion

*Zhangixalusnigropunctatus* was first recorded in Weining District, Guizhou Province, China by Liu et al. (1962) in 1962. Based Orlov et al. (2001) study of a series of specimens collected from Fan Si Pan Mountain, in the northern part of the Hoang Lien Mountains, Sa Pa district, Lao Cai Province, they suggested that *Z.nigropunctatus* might be a junior synonym of *Z.dorsoviridis*, a species originally described by Bourret (1937) from Sa Pa District, Lao Cai Province, Vietnam. Li et al. (2012b) investigated the phylogenetic relationships of the genus *Rhacophorus* sensu lato and showed that a specimen identified as *Z.nigropunctatus* in the collection of Orlov et al. (2001; ROM 38011) was nested in the same subclade as *Z.nigropunctatus* from China (Weining, Guizhou) (Pan et al. 2024). Although the genetic distance between them was greater than 5% and there was no specimen description, Orlov et al. (2001) concluded that both *Z.dorsoviridis* and *Z.nigropunctatus* occur in Sa Pa, Lao Cai Province (see also Pan et al. 2017).

The phylogenetic results of our study reveal that the specimen JX219427 from (ROM 38011; Orlov et al. 2001), which was originally classified as belonging in the *Z.nigropunctatus* clade of Li et al. (2012b), is in fact *Z.thaoae* with a genetic distance of only 0.19 compared with the three other newly found specimens. Furthermore, *Z.thaoae*, *Z.dorsoviridis*, and *Z.nigropunctatus* show high genetic distance of at least 5.5%. This large genetic distance makes it unlikely that by using genetic data, the taxonomic assignment of a specimen into the three aforementioned species can be confused. Although molecular data indicates that ROM 38011 belongs to *Z.thaoae*, detailed morphological data to support such assignment is not available; neither Orlov et al. (2001) nor Li et al. (2012b) provided a detailed description of that specimen, and Orlov et al. (2001) did not provide a figure of ROM 38011. However, Orlov et al. (2001), noted that ROM 38011 had an “obviously darkened” median vocal sac which differed from the other specimens they examined.

Overall, there is evidence that the specimen ROM 38011 had been misidentified as either *Z.dorsoviridis* or *Z.nigropunctatus* and that *Z.thaoae* is a valid new taxon. Based on our reclassification of ROM 38011 as *Z.thaoae* and lack of other known records, we herein formally exclude *Z.nigropunctatus* from the amphibian fauna of Vietnam. *Zhangixalusnigropunctatus* is only known from Yunnan Province (Pingbian), Anhui Province (Yuei), Guizhou Province, (Weining and Leishan), and Hunan Province (Sangzhi and Chengbu), China (Li et al. 2012b; Pan et al. 2017, 2024).

In addition to the new species described in this paper, Nguyen et al. (2020) previously described *Z.jodiae* and assigned earlier records of *Z.dorsoviridis* from Ha Giang Province to *Z.jodiae*. Thus, two species belonging to the *Z.chenfui* group of green treefrogs, *Z.jodiae* and *Zhangixalusthaoae*, are currently known from Vietnam.

## Supplementary Material

XML Treatment for Zhangixalus thaoae

## ﻿Additional information

### Author ORCIDs

Tao Thien Nguyen https://orcid.org/0000-0002-5640-4536

Huy Hoang Nguyen https://orcid.org/0000-0002-6284-5813

Hoa Thi Ninh https://orcid.org/0009-0002-8133-432X

Linh Tu Hoang Le https://orcid.org/0009-0003-0887-2786

Hai Tuan Bui https://orcid.org/0000-0003-4065-7229

Nikolai Orlov https://orcid.org/0000-0003-4401-348X

Chung Van Hoang https://orcid.org/0000-0002-0709-974X

Thomas Ziegler https://orcid.org/0000-0002-4797-609X

### Data availability

All of the data that support the findings of this study are available in the main text.
